# A corrective prescription for GISAXS

**DOI:** 10.1107/S2052252518015087

**Published:** 2018-10-31

**Authors:** Joseph Strzalka

**Affiliations:** a Argonne National Laboratory, X-ray Science Division, 9700 S. Cass Avenue, Argonne, IL 60439, USA

**Keywords:** X-ray scattering, GISAXS, image healing, reconstruction, distorted-wave Born approximation

## Abstract

A new ‘unwarping’ algorithm presented by Liu and Yager in this issue constructs SAXS data consistent with experimental GISAXS data, eliminating many of the complications arising in GISAXS that are commonly modeled within the framework of the distorted-wave Born approximation. The method promises to open new pathways for processing, modeling and analyzing GISAXS data using techniques developed for SAXS.

Winston Churchill famously remarked that democracy is the worst form of government, except for all the others. Similarly, in the thin-film community, grazing-incidence small-angle X-ray scattering (GISAXS) is the worst form of characterization with many flaws that make it difficult to interpret, and yet at the same time it is also considered the most powerful technique available for studying nanostructure at surfaces and interfaces. In this issue of **IUCrJ**, Liu and Yager present an algorithm for simplifying complicated GISAXS data and obtaining the equivalent of transmission SAXS data consistent with the GISAXS results (Liu & Yager, 2018 ▸). Effectively, their ‘unwarping’ algorithm is a prescription for correcting GISAXS vision into the clear picture viewed by SAXS.

GISAXS plays a vital role for characterizing the structure of the thin films that many technologies rely on to economically cover large areas: photovoltaics, organic electronics, directed self-assembly for advanced lithography, *etc*. (Sinha *et al.*, 1988 ▸; Renaud *et al.*, 2009 ▸). The technique depends upon the total external reflection of X-rays from matter at small incident angles, which favors probing a thin film or the surface layers of a sample while suppressing scattering from the underlying substrate. However, the same aspects of the GISAXS technique that result in a strong signal make that signal difficult to interpret. Multiple scattering effects result in overlapping diffraction patterns centered on the direct beam and on the specularly reflected beam. Features shift due to the refraction of the incident and reflected beams. While all of these effects can be described in the framework of the distorted-wave Born approximation (DWBA) (Sinha *et al.*, 1988 ▸), the mathematics can be daunting and time consuming. The algorithm devised by the team from Brookhaven is a strategy for eliminating these distortions and obtaining the SAXS pattern consistent with the observed GISAXS data, opening a range of opportunities for GISAXS data analysis and making thin-film characterization more accessible.

The procedure is an iterative algorithm that starts from an initial guess for a SAXS pattern. By applying the DWBA to this trial function, the algorithm generates corresponding GISAXS patterns, compares them to the target data acquired at multiple incident angles, and refines this trial function until its DWBA-derived GISAXS patterns match the experimental data. Astute readers will point out that application of the DWBA requires knowledge of the transmission and reflection coefficients for the sample, typically measured by X-ray reflectivity (XRR). While XRR measurements require some not insignificant effort, extensive testing shows that the algorithm is not very sensitive to the exact values of the reflection and transmission coefficients and converges to the same SAXS pattern for reasonable estimates of the reflectivity based on prior knowledge of the sample’s layered structure. The results of the algorithm (see Fig. 1 ▸) can be verified against grazing-transmission small-angle-scattering data (Lu *et al.*, 2013 ▸; Mahadevapuram *et al.*, 2013 ▸), although, as the authors point out, the undistorted data from their algorithm, since it is reconstructed from GISAXS data, possesses a superior signal-to-noise ratio to the GTSAXS data.

The authors show that while the algorithm converges for any starting function, even random noise, the speed with which convergence occurs is much faster for well selected initial functions. In order to arrive at the most efficient trial function, the authors have developed another noteworthy algorithm to determine the relative weighting of the transmission and reflection channels in the GISAXS pattern. As is shown in the DWBA, the GISAXS pattern results mainly from the superposition of these two so-called channels, which are the diffraction patterns centered on the direct beam and on the specularly reflected beam, respectively. By means of a clever parameterization, the authors show that the trick to making an effective initial guess for the undistorted scattering amounts to determining the ratio between the signal from these two channels. Using this ratio and the known separation between the two channels calculated for the refraction from the sample, the scattering from each channel can be calculated from the observed GISAXS pattern. Since either channel could be used to predict the observed GISAXS signal independently, only the correct ratio will result in self-consistent predictions for the GISAXS pattern from either channel. This procedure converges in just a handful of iterations to the optimal weighting ratio, which in turn yields the best initial trial function for making the overall algorithm computationally efficient.

While GISAXS and SAXS have always been closely related techniques, the unwarping algorithm provides a way to make all the models, methods of data analysis, and data-analysis pipelines developed for SAXS applicable to GISAXS data. While it is expected that the algorithm may break down when treating data from perfect, monodisperse samples illuminated by strongly coherent X-rays, the range of cases where it could be successfully applied is still potentially very large. With its X-ray vision now corrected, the thin-film community should find its progress accelerating.

## Figures and Tables

**Figure 1:**
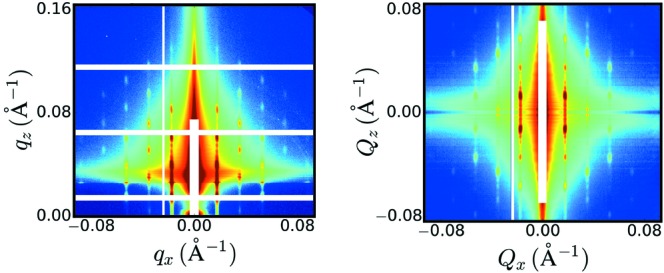
Correcting X-ray vision of thin films: the unwarping algorithm by Liu and Yager takes experimental GISAXS data (left), and generates a corresponding SAXS pattern (right). [Figure adapted from Liu & Yager (2018 ▸) with permission.]
